# Novel Chikungunya Virus Variant in Travelers Returning from Indian Ocean Islands

**DOI:** 10.3201/eid1210.060610

**Published:** 2006-10

**Authors:** Philippe Parola, Xavier de Lamballerie, Jacques Jourdan, Clarisse Rovery, Véronique Vaillant, Philippe Minodier, Philippe Brouqui, Antoine Flahault, Didier Raoult, Rémi N. Charrel

**Affiliations:** * Hôpital Nord, Marseilles, France;; † Unité des Rickettsies, Marseilles, France;; ‡ Fédération de Microbiologie Clinique Hôpital de la Timone, Marseilles, France;; § Unité des Virus Emergents, Faculté de Médecine, Marseilles, France;; ¶ Centre Hospitalier Universitaire, Nîmes, France;; # Institut de Veille Sanitaire, Saint-Maurice, France;; ** Université Pierre et Marie Curie, Paris, France

**Keywords:** alphavirus, arbovirus, chikungunya, Indian Ocean, research

## Abstract

Chikungunya virus (CHIKV) emerged in Indian Ocean islands in 2005 and is causing an ongoing outbreak that involves >260,000 patients, including travelers returning home from these islands. We investigated cases in 4 patients returning from Mayotte and Reunion Islands with CHIKV infection and a nurse infected in metropolitan France after direct contact with the blood of a traveler. Four patients had tenosynovitis and pain at wrist pressure, and 1 had life-threatening manifestations. Four CHIKV strains were isolated, including 1 from the patient with the autochthonous case. The complete genomic sequence identified a new CHIKV variant emerging from the East/central African evolutionary lineage. Aedes albopictus, the implicated vector of CHIKV in Indian Ocean islands, has dispersed worldwide in recent decades. High viral loads in patients returning from Indian Ocean islands to countries where Ae. albopictus is prevalent may be a source of epidemics.

## Abstract 2

*Aedes albopictus* may cause epidemics when infected persons travel to areas where vectors are prevalent.

Human pandemics and emerging infectious diseases such as influenza, HIV, dengue hemorrhagic fever, West Nile encephalitis, and possibly severe acute respiratory syndrome have been attributed to the ability of RNA viruses to evolve rapidly and expand their vector or host range (*1*). To date, most Western countries have escaped much of the health problems that RNA arboviruses inflict on humans in the tropics. However, recent events suggest that this situation may be changing (*2*). The emergence of West Nile virus in 1999 in the United States and its subsequent rapid spread demonstrated that arboviruses are still a threat, even in temperate, industrialized countries. West Nile fever has become the dominant vectorborne viral disease in the United States, with >20,000 reported human cases, 770 deaths, and an estimated 215,000 illnesses during the past 7 years (*3*).

A recent outbreak of chikungunya fever in the islands of the Indian Ocean has drawn attention to chikungunya virus (CHIKV, genus Alphavirus, family Togaviridae) (*4*), first identified in the 1950s in Africa. There, it is maintained in a sylvatic cycle involving wild primates and forest-dwelling Aedes mosquitoes that resembles the epidemiologic cycle of yellow fever virus (*5**–**7*). CHIKV has since been associated with the urban Aedes aegypti mosquito (possibly supplemented by Ae. albopictus) in Asian countries in an epidemiologic cycle resembling that of dengue and characterized by the absence of an animal reservoir, direct human-to-human transmission by urban mosquitoes, and the potential for major epidemics (*8**–**11*). At the beginning of 2005, an outbreak of chikungunya fever was observed in the southeastern islands of the Indian Ocean. The epidemic was most noticeable in urban and semiurban areas of the Comoros Islands, where >5,000 cases have been reported. Thereafter the virus has circulated in other islands, including Reunion and Mayotte (2 French territories), Mauritius, the Seychelles, and Madagascar (*12**–**15*). The population of these islands is >22 million (*16*). At the beginning of 2006, Reunion was experiencing an explosive outbreak. On June 1, an estimated 264,000 CHIKV infections (in a population of 770,000) were reported; 237 death certificates mentioned CHIKV as the possible cause of death (*17*). The implicated vector in this outbreak is Ae. albopictus, the Asian tiger mosquito (*12**,**17*). This mosquito was originally indigenous to Southeast Asia, the Western Pacific, and the Indian Ocean but has recently spread to Africa, the Middle East, Europe and the Americas, mainly because of transportation of dormant eggs in tires (*10*).

The Indian Ocean islands are popular tourist destinations. According to the World Organisation of Tourism, in 2002, some 719,000 tourists arrived in Mauritius, 432,000 in Reunion, 139,000 in Madagascar, and 122,000 in the Seychelles (*18*). In 2004, an estimated 1,474,218 persons traveled from Madagascar (153,766), Mauritius (657,312), Mayotte (63,372) Reunion (498,388), and Seychelles (101,380) to the European mainland (*19*). In addition, hundreds of cases of CHIKV infections have been reported in India and Malaysia (*19*). Recently, CHIKV-infected travelers returned home to countries where competent vectors are indigenous (*19*), which raises serious concern for potential disease spread. We describe 4 patients who returned to southern France, where the Asian tiger mosquito has been established since 2005, with CHIKV infection. One of them was the source of an autochthonous nosocomial infection in a nurse in metropolitan France.

## Patients

**Figure 1:**
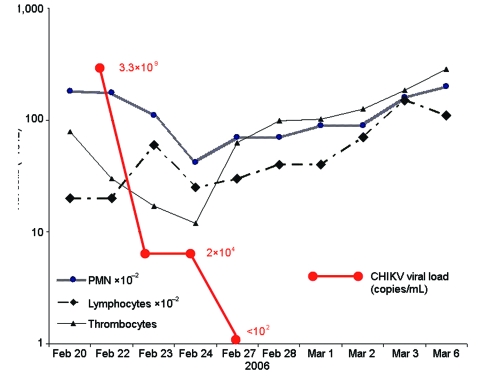
Evolution of viral load and blood cell counts in a 73-year-old man who had returned from Reunion during the acute phase of chikungunya virus (CHIKV) infection. PMN, polymorphonuclear leukocytes.

**Figure 2:**
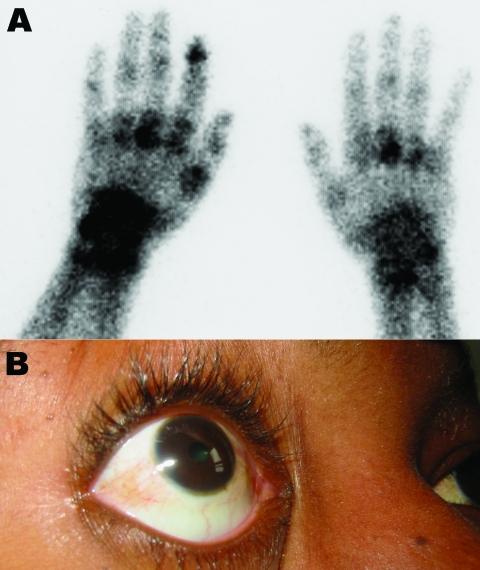
Clinical findings in patients. A) Bone scintigraphy of the wrists and hands showing an intense focus of technetium-99m–labeled methylene diphosphonate tracer uptake, particularly on the left side in the left metacarpophalangeal, wrist, and the first distal interphalangeal joints in a 73-year-old man who returned from Reunion with a severe viremic chikungunya virus (CHIKV) infection. B) Conjunctivitis in a 31-year-old woman who returned from Mayotte, French Comoros, with a severe viremic CHIKV infection.

Patient 1, a 73-year-old man, returned from Reunion on February 17, 2006. His medical history included type 2 diabetes mellitus and oral treatment with imatinib for an intestinal stromal tumor with liver metastasis that was surgically removed in 2004. He was admitted for a 3-day history of fever (temperature 38.5°C) and arthralgia. Clinical signs included asthenia, anorexia, nausea, myalgia, headache, bilateral conjunctivitis, and arthralgia that was particularly intense in shoulders and elbows, left ankle, and left wrists, which suggested tenosynovitis. Purpuric lesions of the legs and intense pain in response to pressure on the left wrist were noticed. Laboratory findings showed severe pancytopenia, including reduced platelet count (17×109 cells/L), reduced leukocyte count (0.7×109 cells/L), and nonregenerative anemia (hemoglobin 9.5 g/dL, 10×109 reticulocytes/L) (Figure 1). Blood chemistry values included raised enzymes: 1,858 IU/L lactate dehydrogenase (LDH), 283 IU/L aspartate aminotransferase (AST), 210 IU/L alanine aminotransferase (ALT), 672 IU/L creatine phosphokinase, and 84 IU/L γ-glutamyl transaminase. Bone marrow smear was cell-rich; granulocytic cells were predominant and hemophagocytosis was limited. Escherichia coli septicemia occurred during the neutropenic period and was successfully treated with ceftriaxone. The patient recovered slowly after a 5-day regimen of high-dose intravenous immunoglobulin, but intensive joint pain persisted. On March 2, bone scintigraphy showed diffuse inflammation of all joints, specifically at the left shoulder, wrist, and hand (Figure 2A).

Patient 2 was a 5-month-old Canadian child who was hospitalized in Marseilles on February 24, 3 days after returning from Reunion and a few days before traveling to Canada. Symptoms included fever and a macular rash; laboratory findings showed elevated serum C-reactive protein (CRP) (37 mg/L) and AST (59 IU/L). Blood cell counts showed no abnormalities. He was discharged, and his fever slowly abated.

Patient 3 was a 31-year-old woman who had been living in Marseilles since 1987 and who had returned from Mayotte, a French Comoros island, on February 28, 2006. Two days later, she was admitted with fever (temperature 39.2°C), nausea, myalgia, lumbar pain, headache, bilateral conjunctivitis (Figure 2B), and severe bilateral arthralgia (shoulders, knees, and particularly ankles, elbows, wrists, and fingers). Exquisite pain was noted when pressure was applied to the right wrist. Laboratory findings included negative blood smears for malaria, anemia (hemoglobin 10.8 g/dL), and lymphopenia (0.6×109 cells/L). Blood chemistry values were within normal limits, except for raised enzymes (177 IU/L AST, 116 IU/L ALT, 780 IU/L LDH), hypocholesterolemia (3.5 mmol/L), and CRP (64 mg/L). Fever disappeared at day 4, and the patient was discharged, although still in pain.

In January 2006, a 75-year-old female patient (patient 4) returned from Reunion with a sudden-onset fever, asthenia, arthralgia with wrist pain, and diarrhea. Two days later, blood specimens were collected at the patient's home by a 60-year-old female nurse (patient 5). At this time, patient 4 had high fever (temperature 40°C). Three days later, fever, skin rash, and arthralgia with pain at wrist pressure developed in the nurse. Interview and examination did not show recent travel abroad, mosquito bite, accidental skin puncture during blood sampling, skin lesion, or eczema. The nurse washed her hands with a hydroalcoholic solution before and after drawing blood, but she did not wear gloves. She noted direct contact with patient 4's blood during hemostasis.

## Methods

Acute-phase sera obtained from patients 1, 2, 3, and 5 were tested for CHIKV RNA through a quantitative real-time reverse transcription (RT)-PCR test (*20*) and used for partial E1 gene sequence determination and virus isolation in Vero E6 cells. Viral loads were estimated by comparative analysis by using threshold values obtained with serial dilutions of a 450-nucleotide (nt) in vitro transcribed RNA, quantified spectrophotometrically as previously reported (*21*), encompassing the target region (detailed protocol available on request). CHIKV-specific immunoglobulin G (IgG) and IgM were tested in acute- and convalescent-phase sera by an indirect fluorescent antibody test with a hyperimmune mouse ascitic fluid prepared against CHIKV Ross strain in conjunction with a fluorescein isothiocyanate–conjugated goat anti-human IgG (Fluoline G, bioMérieux, Marcy l'Etoile, France) as previously described (*22*). Primers used to completely sequence strain LR2006-OPY1 (first passage) were designed from CHIKV and o'nyong-nyong virus sequences retrieved from GenBank. Sequence reconstruction was performed with Sequencer software program (Gene Codes Corp., Ann Arbor, MI, USA). E1 gene sequences (1,225 nt) were generated by using primers CHIKE1S1, 7 ACATCACGTGCGAGTACAAAAC and CHIKE1R1, TCTCTTAAGGGRCACATATACC. Phylogenetic and evolution studies were performed with E1 sequences as previously described (*23*) with ClustalX version 1.81 (*24*) for sequence alignments and MEGA version 2.1 (*25*) for phylogenetic analyses.

## Results

CHIKV infection was diagnosed by positive RT-PCR in acute-phase sera (viral loads of 3.3×109, 1.0×107, 4.2×108, and 2.0×108 copies/mL in patients 1, 2, 3, and 5, respectively). Patient 1's viral load decreased to 2×104 copies/mL at day 2 and 3 and was negative at day 4 (Figure 1). Patient 3's viral load decreased to 7×105 copies/mL at day 4, 3×104 copies/mL at day 7, and was negative at day 8. Infection was also diagnosed by seroconversion with no antibody in acute-phase serum and IgM and IgG in convalescent-phase serum or isolated IgM in acute-phase serum (patient 2, no convalescent-phase serum) and by virus isolation from acute-phase serum (isolates LR2006-OPY1, LR2006-OPY2, MCF2006-OPY4, and GARD2006-OPY6 for patients 1, 2, 3, and 5, respectively).

**Figure 3:**
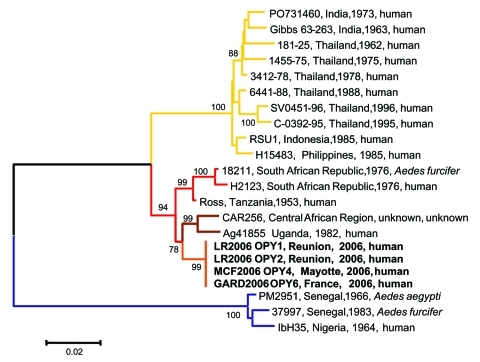
Phylogenetic analysis of chikungunya virus (CHIKV) isolates based on a 1,044-nucleotide (nt) fragment between nt 10243 and 11286 (numbered after strain Ross [accession no. AF490259]) in the E1 gene. Distances and groupings between the 3 Indian Ocean isolates and 18 isolates previously characterized (*23*) were determined by the Jukes-Cantor algorithm and neighbor-joining method with the MEGA software program (*25*). Bootstrap values >75% are indicated and correspond to 500 replications. The main evolutionary lineages, East/Central African (brown), eastern/southern Africa (red), West African (blue), and Asian (yellow), are indicated. The Indian Ocean sublineage is indicated in orange. **Boldface** indicates sequence determined in this study.

A 1,044-nt sequence of the E1 gene was used for comparative genetic analysis of sequences from Indian Ocean CHIKV and reference strains from diverse geographic and temporal origins (*23*). Indian Ocean strains formed a sublineage that is closely related to but distinct from viruses belonging to the East/Central African evolutionary lineage (Figure 3). The E1 nucleotide sequences of the 4 CHIKV isolates from 2006 (GenBank accession nos. DQ451149–DQ451151) were 100% identical, while ranges of 2.1%–3.3%, 14.8%–15.8%, and 5.5%–6.4% of nucleotide divergence were observed when compared with isolates from Central/East Africa, West Africa, and Asia, respectively.

Phylogenetic analysis and high bootstrap values indicated that the 4 Indian Ocean strains had a common ancestor, constituted a new sublineage that is distinct from the 4 lineages previously recognized, and most likely emerged recently to cause the regional outbreak (*26*). Two mutations, which are apparently specific for strains circulating in Reunion and Mayotte, consist of A1028→V and D1086→E substitutions with reference to all other CHIKV strains characterized to date, regardless of evolutionary lineage (amino acid positions refer to the sequence of the original Ross isolate, accession no. AF490259). A and D residues are also found in available sequences of o'nyong-nyong virus, another alphavirus distantly related to CHIKV. The complete coding sequence of the strain recovered from patient 1 (LR2006 OPY1) was determined and deposited in GenBank (accession no. DQ443544). Comparative analysis with the 3 other full-length sequences available for CHIKV showed that the overall nucleotide distances were 2.7% (1.4% amino acid) with East/Central strains and 14.8% (4.4% amino acid) with West African strains (no full-length sequence was available for CHIKV strains from Asia).

Diagnosis of a CHIKV infection in patient 5 was based on clinical (typical chikungunya fever signs and symptoms), epidemiologic (taking care of a patient who was returning from Reunion at the time of the outbreak), and serologic (IgM- to IgG-specific conversion) evidence. After contact with patient 4, CHIKV infection of patient 5 was shown by RT-PCR, virus isolation, and seroconversion as reported above.

## Discussion

Visitors to tropical countries and workers migrating to Western countries may serve as sentinels for the global dissemination and emergence of viral, bacterial, and parasitic agents that affect local populations in the tropics (*27*). As an example, the outbreak of chikungunya fever in 2005 in the Comoros Islands led to the first documented CHIKV infections imported into France by travelers who had visited the islands (*28*). The patients reported here had typical clinical signs and symptoms of CHIKV infection, including febrile polyarthritis with papular, macular, or purpuric rashes; hemorrhagic manifestations are, however, uncommon. Another characteristic was severe arthralgia accompanied by extremely painful arthritis and tenosynovitis and specific pain when pressure was applied to the wrists. Severe thrombocytopenia and neutropenia complicated by septicemia developed in patient 1. In Reunion, complicated clinical syndromes have been reported mainly in newborns, the elderly, and disabled patients (*17*). Previous studies have shown that a proportion of CHIKV-infected patients remain in severe pain for months (*29**,**30*). In patient 1, and in subsequent patients in our units, we also observed late-to-chronic CHIKV rheumatic manifestations, particularly subacute tenosynovitis of the wrists, hands, and ankles (*31*).

Virologic studies identified a new CHIKV variant in patients infected from Mayotte and Reunion, with specific amino acid mutations in the E1 gene; these findings may be useful in the future for tracing dissemination of the virus. The genetic heterogeneity between this variant and other strains in the East/Central Africa lineage has a magnitude equivalent to or higher than that observed between strains of West Nile virus circulating in southern Europe and the Middle East for the past 4 decades and which emerged and dispersed in the United States in 1999. Phylogenetic analysis showed that this variant originated from the East/Central African lineage of CHIKV and emerged recently, in accordance with the observed epidemic pattern. High viremia was observed in this study (up to 3.3×109 copies/mL), which enabled virus isolation from all viremic patients. We used the same diagnostic system as reported by Pastorino et al. (*20*), but viral load was quantified with RNA that encompassed the target region by using a method similar to that described previously (*21*). RNA was then quantified by spectrophotometry, diluted serially from 100 to 10–14; the 4 highest dilutions that provided a positive result were included in each diagnostic experiment. The quantification was performed with an in vitro–transcribed RNA based on a strain that was genetically distinct from those circulating in Indian Ocean islands. However, in the region targeted by the real-time PCR assay, 206 of 209 sites are conserved between the sequence of Ross strain and strains circulating in 2006 in the Indian Ocean region. Moreover, no mismatch exists in the sequence targeted by the primers or the probe. This finding suggests that the quantification of viral loads, although approximate, is a good reflection of biologic reality.

Although viral loads up to 109 have been reported in dengue virus infection, such levels of viremia are uncommon in arthropodborne viral diseases such as dengue fever and West Nile virus infection (*32**–**34*). In the absence of published data, we cannot determine whether high viral loads are common during CHIKV infection or specific to Indian Ocean strains. They may account for the autochthonous case reported here. Patient 5's CHIKV infection was associated with direct contact with the blood of patient 4; in addition, Aedes spp. do not generally bite in southern France during the winter. Virus transmission through direct contact with highly viremic blood appears to be the most plausible hypothesis to explain this autochtonous CHIKV infection. Whether the skin was intact when contact with infected blood occurred could not be clearly determined.

A major determinant of outbreak dynamics is the ecologic cycle of the virus and its vector. This cycle in Reunion probably follows a denguelike model characterized by the absence of an animal reservoir and the ability to spread rapidly among humans through peridomestic mosquito bites. We suspect that direct transmission to healthcare workers may have also occurred in disease-endemic countries, but it was not identified.

**Figure 4:**
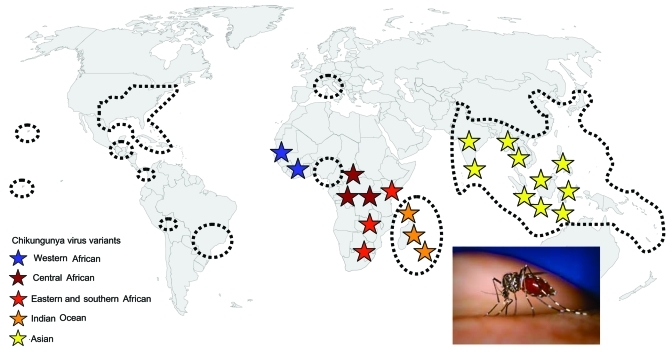
Estimated global distribution of *Aedes albopictus* (areas enclosed in dotted lines) and distribution of chikungunya virus (stars) from western Africa to southeastern Asia, including the Indian Ocean variant responsible for the 2006 outbreak. The color of the stars reflects the main evolutionary lineages shown in Figure 3. *Ae. albopictus* photograph courtesy of James Gathany, Centers for Disease Control and Prevention.

Because dengue virus does not require a nonhuman vertebrate reservoir, it was able to disseminate in regions where competent vectors were present. CHIKV may become similarly globalized. Since the 1980s, Ae. albopictus has spread worldwide; it reached the United States in 1985, Brazil in 1986, Central America in 1988, and Africa in 1992 (Figure 4) (*10*). In Europe, it was identified in Albania in 1979 and in Italy in 1991, where it has become established (*10*). In France, it was reported for the first time in 1999 (*35*). It has also been introduced into several other European countries, including Belgium, Bosnia and Herzegovina, Croatia, Greece, the Netherlands, Serbia and Montenegro, Slovenia, Spain, and Switzerland (*19*). In 2004, Ae. albopictus was identified in several areas of the French Riviera, only 200 km from Marseilles, where 3 of our viremic patients were hospitalized (*36*). Although Ae. albopictus is active year-round in tropical (e.g., Central America) and subtropical (e.g., Gulf Coast) latitudes, it overwinters in its egg stage in the colder latitudes of the Northern Hemisphere. Therefore, risk for CHIKV spread is probably absent during colder months in Europe. However, in Italy, Ae. albopictus is active from February through December, with a peak in August and September (*37*). Whether ecologic conditions during the period of Ae. albopictus activity in southern Europe and North America support the development of a productive and persistent viral cycle in local vector populations is unknown. Furthermore, we do not know whether Ae. albopictus can transmit vertically and thus transfer CHIKV to the next generation (and the next season), as has been shown for dengue virus (*10*). If viremic patients arrive in Italy, France, or elsewhere in southern Europe during the summer, however, they could cause a European outbreak. The risk may also exist in the Americas, where the Asian tiger mosquito is prevalent.

## Conclusion

In 1 year, >250,000 persons have been infected by a new CHIKV variant on the Indian Ocean islands. Although major differences in the fitness or virulence of CHIKV may be associated with minor genetic differences, the most likely explanation for this devastating outbreak is the penetration into a region where the population is immunologically naive for CHIKV and where Ae. albopictus proliferates. As this outbreak has spread, we believe this type of outbreak could occur in other regions of the world where competent vectors are prevalent. Because of high viremia, the virus could also be directly transmitted to healthcare workers. We must be prepared for the possibility of similar arboviral epidemics in such places.
